# Impact of Age and Gender on Survival of Glioblastoma Multiforme Patients: A Multicenter Retrospective Study

**DOI:** 10.1002/cnr2.70050

**Published:** 2024-11-07

**Authors:** Zoheir Reihanian, Elahe Abbaspour, Nooshin Zaresharifi, Sahand Karimzadhagh, Maral Mahmoudalinejad, Ainaz Sourati, Mohaya Farzin, Habib EslamiKenarsari

**Affiliations:** ^1^ Department of Neurosurgery, Poursina Hospital Guilan University of Medical Sciences Rasht Iran; ^2^ Clinical Research Development Unit of Poursina Hospital Guilan University of Medical Sciences Rasht Iran; ^3^ Department of Pathology, Faculty of Medicine Guilan University of Medical Sciences Rasht Iran; ^4^ Department of Internal Medicine, School of Medicine Shahid Beheshti University of Medical Sciences Tehran Iran; ^5^ Department of Radiation Oncology Guilan University of Medical Sciences Rasht Iran; ^6^ Department of Physiology, Razi Clinical Research Development Center Guilan University of Medical Sciences Rasht Iran; ^7^ Research and Technology Guilan University of Medical Sciences Rasht Iran

**Keywords:** brain tumor, GBM, gender, glioblastoma, overall survival, sex difference

## Abstract

**Background:**

Glioblastoma multiforme (GBM) poses a significant health challenge as the most common primary malignancy of the adult central nervous system. Gender‐ and age‐related differences in GBM influence prognosis and treatment complexities. This multicenter retrospective study explores gender and age disparities in GBM patients, investigating their impact on occurrence and survival outcomes.

**Methods:**

This multicenter retrospective study involved GBM patients treated in Guilan Province, Iran. Patients' data, including age, gender, tumor location, and histopathological diagnosis date, was collected from medical records.

**Results:**

In a cohort of 164 GBM patients, the average age was 54.34 ± 14.16 years, with a higher prevalence among men (59.8%) and patients aged ≤ 60 years (64.6%). The tumor sites exhibited overlapping features in 68% of cases, with the frontal and temporal lobes being the most common specific locations. The mean survival was 12.88 ± 14.14 months, one‐year survival of 45%, with women showing significantly higher one‐year survival (60% vs. 40%) and longer mean survival (16.14 ± 17.35 vs. 10.75 ± 11.15 months). Furthermore, Patients ≤ 60 years had significantly higher one‐year survival (75% vs. 35%). In subgroup analysis, women had significantly higher survival rates in patients ≤ 60 years. However, among patients over 60, women exhibited a more pronounced decline in survival rates, with no statistically significant difference between men and women in this age group.

**Conclusion:**

This study highlights that both age and gender significantly affect GBM survival outcomes. Younger patients, particularly women, exhibited better survival rates, while older patients, especially women, showed poorer outcomes. These findings suggest the need to stratify treatment approaches by both age and gender to optimize care and improve survival in GBM patients. Further research is recommended to explore these associations.

## 1 Introduction

Glioblastoma multiforme (GBM) ranks as the most prevalent and highly invasive primary malignancy of the central nervous system(CNS) in adults [1, 2], constituting 57.3% of all gliomas and 48.3% of malignant brain tumors [3, 4]. As the global population ages, the incidence of GBM increases. Older patients being diagnosed with GBM generally face a less favorable prognosis compared to their younger counterparts [5], experiencing a median overall survival (OS) of 9 months, in contrast to the 15‐month OS observed in the general adult population. The management of GBM in older patients can be more complex due to age‐related comorbidities and the potential impact of treatment on their quality of life [6]. Moreover, gender influences GBM onset, with a male‐to‐female ratio of 1.6:1 [7]. Previous studies suggest that females are associated with better outcomes in both adults and children. Although there is some evidence indicating the potential involvement of sex hormones, the exact causes of the observed differences remain unclear [8].

According to the World Health Organization (WHO) classification, glioblastoma multiforme (GBM) is categorized into two subtypes based on genetic characteristics, specifically the presence or absence of isocitrate dehydrogenase (IDH) mutations: IDH‐mutant and IDH‐wild type [9, 10]. These subtypes are referred to as primary (IDH‐wild type) and secondary (IDH‐mutant) GBMs. Primary GBMs generally impact older patients, lack precursor lesions, and are associated with a less favorable prognosis. In contrast, secondary GBMs occur in younger individuals, arise from lower‐grade gliomas, feature IDH mutations, and show a more extended overall survival (OS) [11, 12]. Studies have shown that IDH mutation status significantly influences treatment response, with distinct outcomes observed based on the mutation type. Additionally, methylguanine‐DNA methyltransferase (MGMT) promoter methylation serves as a crucial molecular prognostic factor, predicting the efficacy of alkylating agent therapy [13]. Other predictive factors include clinical parameters, the extent of surgical resection, and tumor imaging characteristics, such as tumor size, location, the presence of necrosis, and surrounding edema [14].

The primary treatment involves comprehensive surgical removal while preserving neurological function and minimizing postoperative complications. Preoperative and intraoperative assessments, encompassing laboratory tests, neuronavigation, intraoperative MRI, and fluorescence‐guided surgery, are pivotal for safe and maximal tumor resection [15, 16]. The treatment protocol extends to postoperative care, including radiotherapy and chemotherapy [15, 16]. This often includes using temozolomide (TMZ), an oral chemotherapy agent with methylating properties [17, 18]. The unfavorable prognosis associated with the tumor comes from its tendency to persist even after surgical resection and adjuvant therapies.

Tumor complete removal is difficult due to the infiltrative tumor growth into the adjacent brain tissue and the brain's vulnerability to surgical interventions, which could lead to functional impairment [19, 20]. Despite advancements in medical care, GBM patients have consistently confronted an unfavorable prognosis in recent years, with a survival rate of less than 7% over 5 years [21], underscoring the persistent challenge of managing this highly aggressive and rapidly progressing malignant tumor. The situation highlights a major challenge in global public health, emphasizing the urgent demand for innovative approaches [22, 23].

Within the context of the challenging survival rates of GBM, this study investigates the factors impacting survival, explicitly age and gender. The study's population consists of individuals who sought medical care at educational and medical institutions of Guilan Province, located in northern Iran, from 2014 to 2018.

## 2 Materials and Methods

### 2.1 Study Design and Setting

This multicenter retrospective study aimed to investigate registered cases of GBM among individuals who underwent medical treatment at educational and medical facilities in Guilan Province, Iran.

### 2.2 Participants and Variables

The study incorporated 164 patients diagnosed with GBM registered from 2014 to 2018, utilizing standardized data collection forms derived from hospital medical records. It is important to note that the diagnostic criteria for GBM have evolved since 2017. However, this study is based on histological characteristics and diagnostic criteria that were in use prior to these updates, specifically focusing on high‐grade glial tumors with palisading necrosis and/or endovascular proliferation. Inclusion criteria encompassed patients with confirmed histopathological and immunohistochemical diagnoses of GBM who received care in Guilan Province during the specified period. Exclusion criteria comprised non‐diagnostic biopsies (e.g., inadequate samples, sampling from non‐tumorous tissue or necrosis only) and insufficient histopathological evidence to confirm a grade 4 glioma. Furthermore, patient information, including age, gender, tumor location, and histopathological diagnosis date, was collected. The overall survival status of each patient was verified using their national ID to cross‐reference death reports. Survival duration, measured in months from the histopathological diagnosis to the time of death, was calculated for deceased individuals.

### 2.3 Ethical Consideration

This study adhered to ethical guidelines, obtaining approval from the Institutional Review Board at Guilan University of Medical Sciences, Iran, before data collection and subsequent analysis (Ethics Approval Code: IR.GUMS.REC.1401.461).

### 2.4 Statistical Analysis

The data were compiled and summarized in a Microsoft Excel (2019) spreadsheet, and subsequent statistical analysis was conducted using SPSS software version 22. Quantitative variables were summarized by mean and standard deviation, while categorical variables were represented through frequency and percentage. The normality of numerical data distribution was assessed through the Kolmogorov–Smirnov test, and variance homogeneity was evaluated using the Levene test. We employed Kaplan–Meier survival curves to determine overall survival rates, and differences between survival curves were compared via the log‐rank test. Additionally, we utilized a Cox regression analysis to investigate the impact of independent variables on overall survival. The Cox proportional hazards regression assumptions were tested using Stata version 18.0 to ensure model appropriateness. Statistical significance was established at a threshold of 0.05, with results considered significant if the *p*‐value was less than 0.05. All reported findings include a 95% confidence interval to ensure accuracy and reliability.

## 3 Result

### 3.1 Characteristics of the Study Population

**FIGURE 1:**
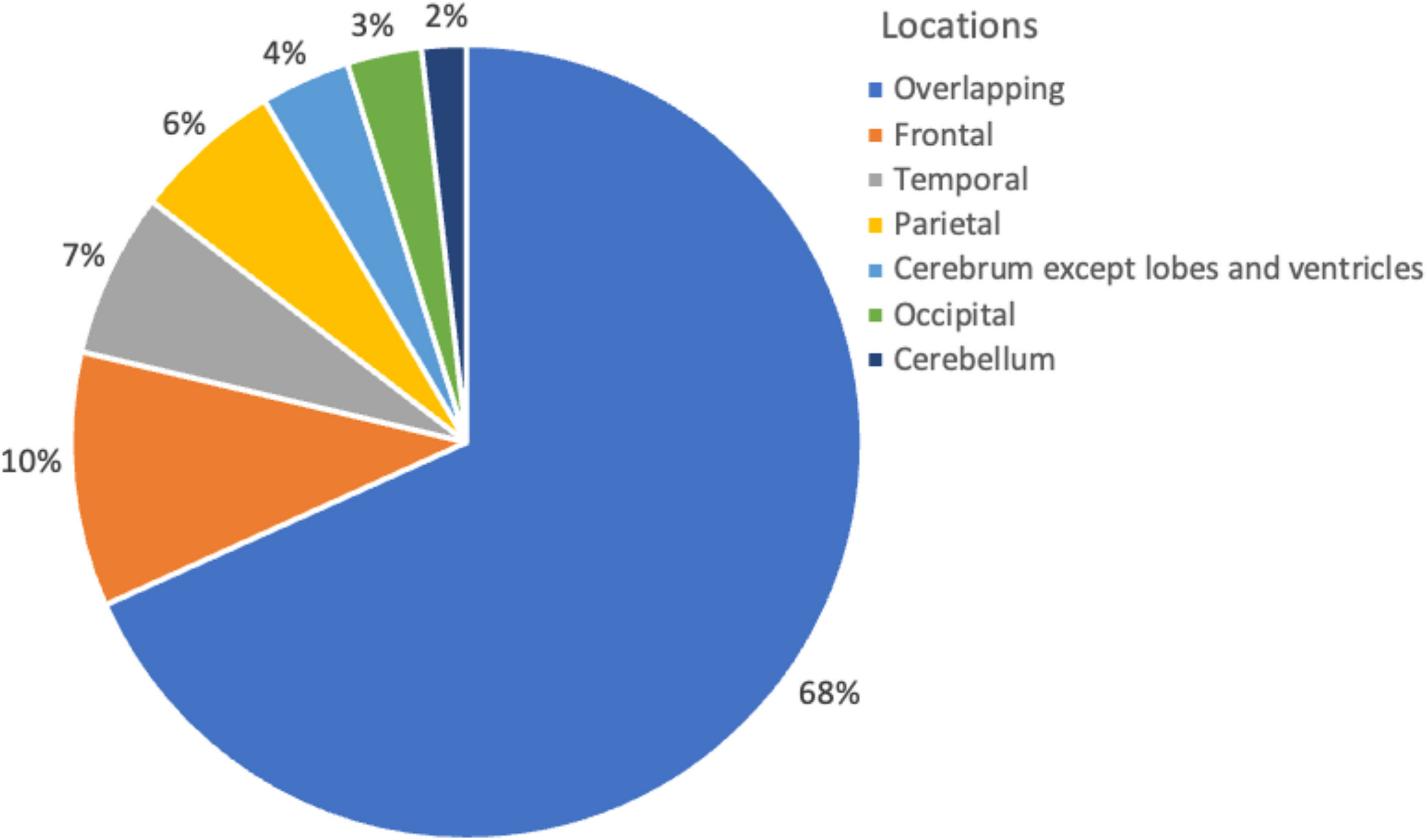
Demonstrating the common locations of glioblastoma within the population.

In our study, we initially identified 284 GBM patients who received care in Guilan Province from 2014 to 2018. However, 120 patients were excluded from the analysis due to incomplete or missing critical data. Consequently, the records of 164 patients were retrospectively analyzed. The study cohort had a mean age of 54.34 ± 14.16 years, ranging from 3 to 82 years. Among these, 106 patients (64.6%) were 60 years or younger, and 98 (59.8%) were male. The higher prevalence in men and patients aged 60 years or younger was statistically significant (*p* < 0.001) (Table 1). Moreover, the tumor site exhibited overlapping characteristics among 112 patients. The most common specific locations were the frontal lobe, observed in 17 cases, the temporal lobe in 11 cases, and the parietal lobe in 10 cases Figure 1.

**TABLE 1:** Characteristics of GBM patients enrolled in the study.

		No. (%)
Sex	Female	66 (40.2)
	Male	98 (59.8)
Age	> 60	58 (35.4)
	≤ 60	106 (64.6)
Location	Overlapping	113 (68.9)
	Frontal	17 (10.3)
	Temporal	10 (6)
	Parietal	10 (6)
	Cerebrum (except lobes and ventricles)	6 (3.6)
	Occipital	5 (3)
	Cerebellum	3 (1.8)

### 3.2 Overall Survival

**FIGURE 2:**
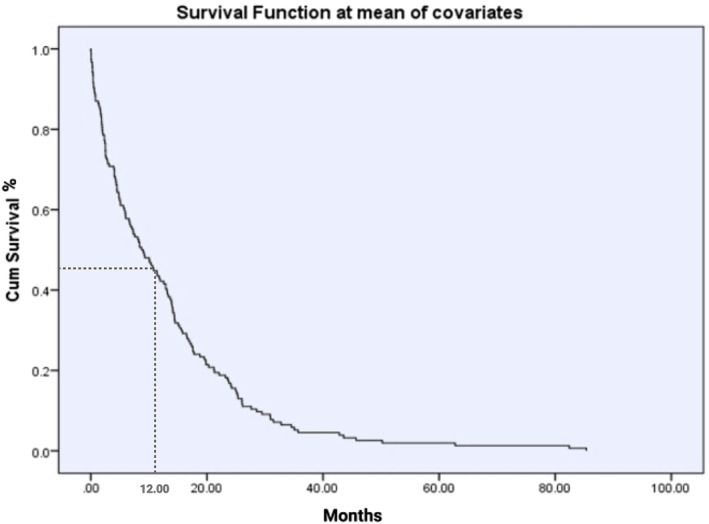
Kaplan–Meier graphs on the survival of the whole study population showing a one‐year survival rate of 45% and median survival time of 12.88 ± 14.14 months for all patients.

**FIGURE 3:**
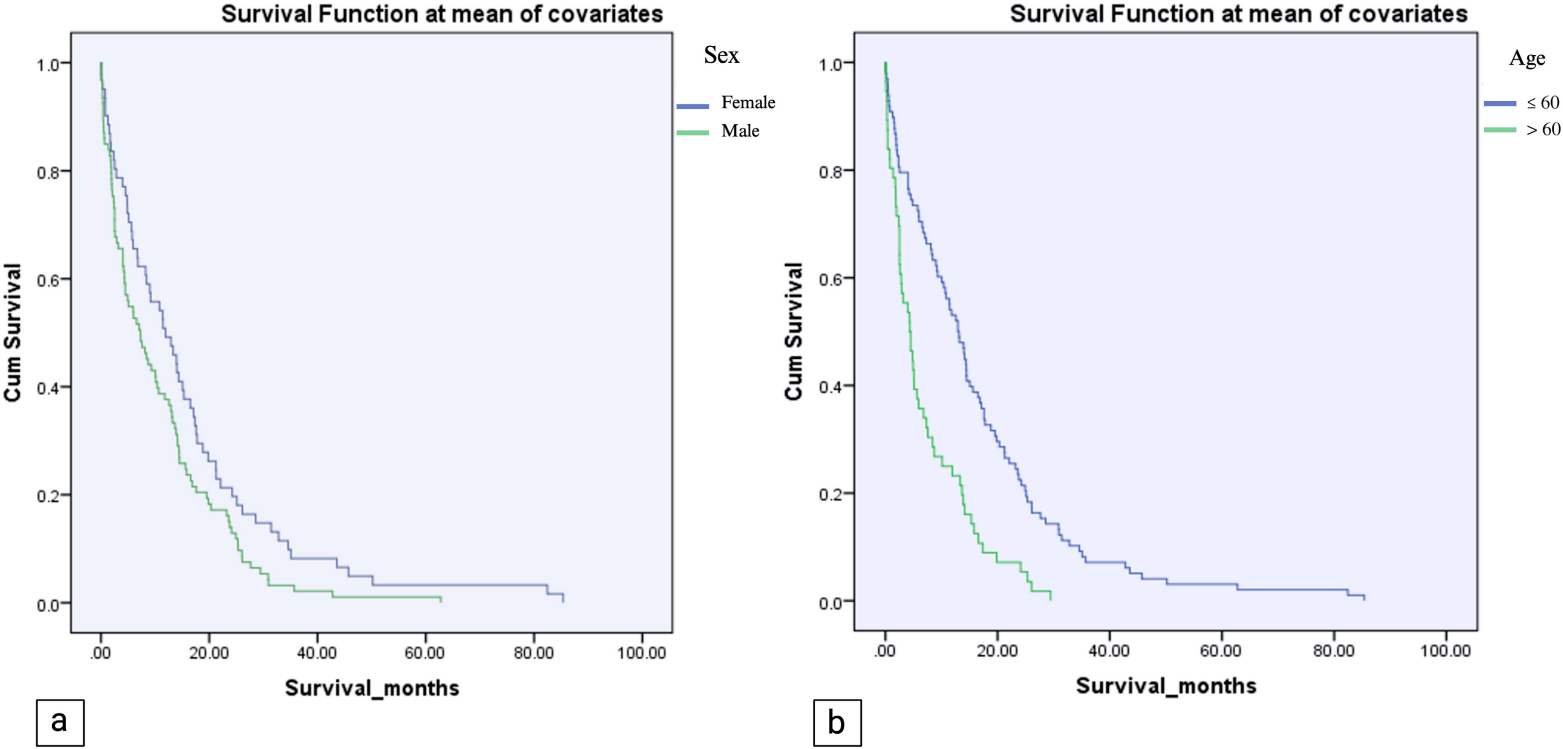
(a) Kaplan–Meier graphs of survival exhibiting a significantly higher survival time in female patients with a median survival of 16.14 ± 17.35 months, in contrast to 10.75 ± 11.15 months in males (*p*‐value: 0.023). (b) Kaplan–Meier graphs of survival show patients aged ≤ 60 years with a significantly higher one‐year survival time (15.9 ± 16.21 months) than patients aged > 60 years (7.48 ± 7.06 months).

The mean survival for the entire cohort was 12.88 ± 14.14 months, spanning from 0 days to 85.37 months. Furthermore, the estimated one‐year survival rate for all patients was 45% (Figure 2). A notable disparity was observed upon gender‐based analysis, with women demonstrating a more favorable one‐year survival rate of 60% (95% CI: 47.8%–72.4%) compared to men at 40% (95% CI: 30.0%–50.1%) with an odds ratio (OR) of 2.32 (95% CI: 1.17–4.63). Additionally, women exhibited significantly longer mean survival time, with an average of 16.14 ± 17.35 months, while men had a mean survival time of 10.75 ± 11.15 months (*p* = 0.023). Individuals over 60 experienced shorter mean survival times than their younger counterparts, with averages of 7.48 ± 7.06 months versus 15.9 ± 16.21 months, respectively. The age‐dependent survival difference was highly statistically significant (*p* < 0.001). Moreover, patients aged 60 or younger demonstrated a significantly higher one‐year survival rate compared to their older counterparts (75% vs. 35%) (Figure 3).

**FIGURE 4:**
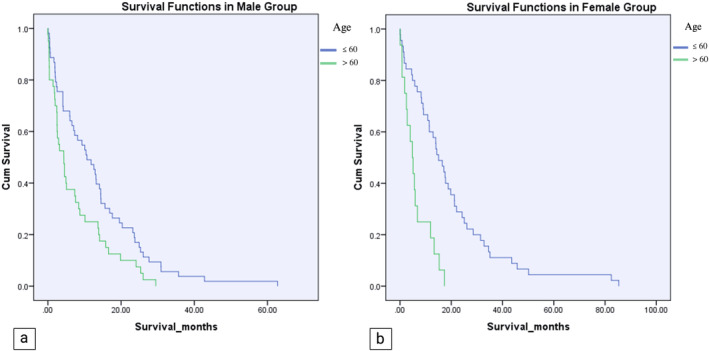
(a) Kaplan–Meier graphs of survival among males. Individuals aged > 60 displayed significantly shorter survival periods than those aged ≤ 60 years (7.41 ± 8.19 vs. 13.27 ± 12.43 months, *p*‐value: 0.009). (b) Kaplan–Meier survival graphs among Females. Females aged > 60 years exhibited markedly reduced survival durations compared to females aged ≤ 60 years (6.18 ± 5.41 vs. 19.68 ± 18.76 months, *p*‐value < 0.001).

**FIGURE 5:**
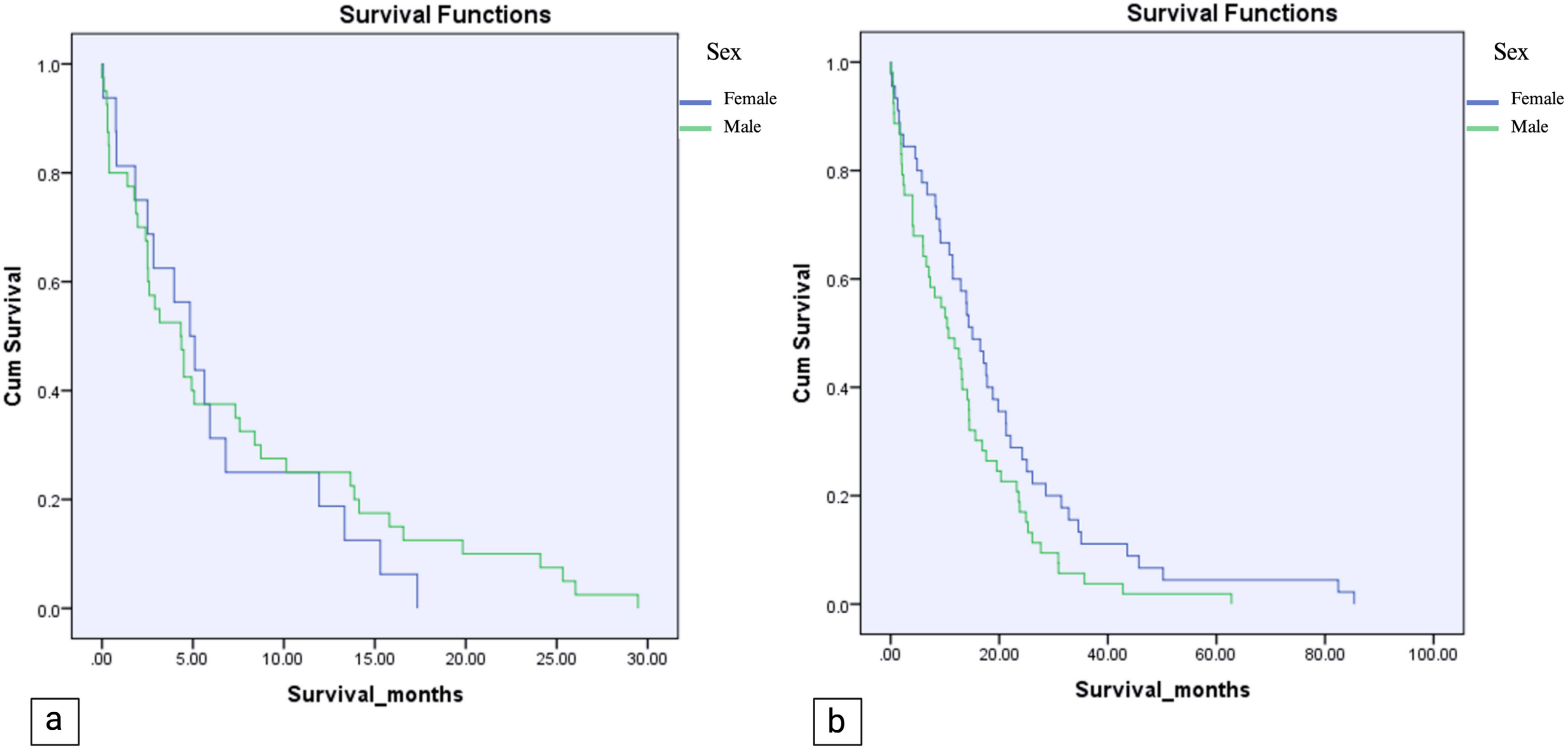
(a) Kaplan–Meier graph of survival among patients aged > 60 displayed no significant difference between men and female (7.41 ± 8.19 months vs. 6.18 ± 5.41 months, *p*‐value: 0.56). (b) Kaplan–Meier survival graph among patients aged ≤ 60 years exhibited markedly reduced survival time in men aged ≤ 60 compared to female aged ≤ 60 years (19.68 ± 18.76 months vs.13.27 ± 12.43 months, *p*‐value < 0.045).

Subgroup analysis based on gender revealed striking differences in survival outcomes. Specifically, women over 60 exhibited markedly reduced survival durations compared to their younger counterparts (6.18 ± 5.41 months vs. 19.68 ± 18.76 months, *p* < 0.001). A similar trend was observed in men over 60 compared to younger males (7.41 ± 8.19 months vs. 13.27 ± 12.43 months, *p* = 0.009) (Figure 4). In the age subgroup analysis, men exhibited significantly lower survival rates than women in patients aged 60 or younger (13.27 ± 5.41 months vs. 19.68 ± 18.76 months, *p* < 0.045). However, no statistically significant difference was observed between males and females aged over 60 (*p* = 0.56). (Figure 5). The Summary of the results is shown in Table 2. Furthermore, In the multivariable Cox proportional hazards model, age was found to significantly affect survival (HR: 1.024, 95% CI: 1.012–1.037, *p* < 0.001), indicating that each additional year of age slightly increased the risk of mortality. In contrast, gender did not show a statistically significant association with survival (HR: 0.782, 95% CI: 0.560–1.091, *p* = 0.148).

**TABLE 2:** Results of the subgroup analysis.

	Group	Subgroup	Mean survival time (month)	*p*
Age		≤ 60 years	16.21 ± 15.9	< 0.001
		> 60 years	7.06 ± 7.48	
Gender‐based	Female	≤ 60 years	19.68 ± 18.76	< 0.001
		> 60 years	6.18 ± 5.41	
		Total	16.14 ± 17.35*	—
	Male	≤ 60 years	13.27 ± 12.43	0.009
		> 60 years	7.41 ± 8.19	
		Total	10.75 ± 11.15*	—

* The *p*‐value comparing total survival between females and males was 0.023.

## 4 Discussion

In this study, we explored the impact of gender and age on the occurrence and survival outcomes of GBM, yielding several important insights. In our cohort, men and patients aged 60 or younger were more frequently affected by GBM. Survival outcomes also varied notably across gender and age groups. Women, in particular, exhibited better overall survival rates, including a higher one‐year survival rate and longer mean survival time compared to men. This gender‐based survival advantage was most evident in women aged 60 or younger, where we observed a significant survival benefit. However, this survival advantage diminished with age. Among patients over 60, women experienced a more pronounced decline in survival, and no significant difference in survival rates was observed between males and females in this older age group. This accelerated decline in survival among older women may be linked to the protective role of gonadal steroid hormones, particularly estradiol, which decreases during menopause, suggesting a complex relationship between gender, age, and survival outcomes in GBM [24].

In line with our findings, prior research has consistently highlighted significant gender disparities in both the incidence and prognosis of gliomas, particularly glioblastoma [25, 26]. Despite extensive investigations on this topic, the biological mechanisms underlying these gender differences in GBM remain partially understood [27, 28]. For instance, Sun et al. [29] suggested that this gender gap could be partially attributed to a higher vulnerability to malignant transformation in male astrocytes when both the p53 and NF1 genes lose their normal functions compared to female astrocytes. Moreover, Khan et al. [30], utilized data from The Cancer Genome Atlas (TCGA) and the Chinese Glioma Genome Atlas (CGGA) to identify molecular markers that may account for gender‐based differences. They discovered that specific autosomal genes such as NOX, FRG1BP, and AL354714.2, along with X‐linked genes such as PUDP, KDM6A, DDX3X, and SYAP1, displayed varying DNA methylation and gene expression profiles in male and female GBM cases.

Furthermore, high expression of estrogen‐related receptor alpha (ERRα) is considered a detrimental factor associated with malignant progression and poorer overall prognosis in various cancer types [31, 32, 33]. In contrast, Hönikl et al.'s study demonstrated that high expression of estrogen receptor alpha (Erα) and aromatase in 60 GBM tissue samples was associated with longer survival times, and treatment with high concentrations of estradiol resulted in reduced tumor cell viability [34]. Despite contradictory findings on estrogen receptor subtypes as prognostic factors, studies suggest a protective role of estradiol (E2), mainly through estrogen receptor beta (Erβ), with varying effects depending on ERβ isoform quantities [28]. While the estrogen‐related pathway has been extensively researched in glioma, it has been challenging to translate this knowledge into practical clinical applications within standard treatment protocols [32].

**FIGURE 6:**
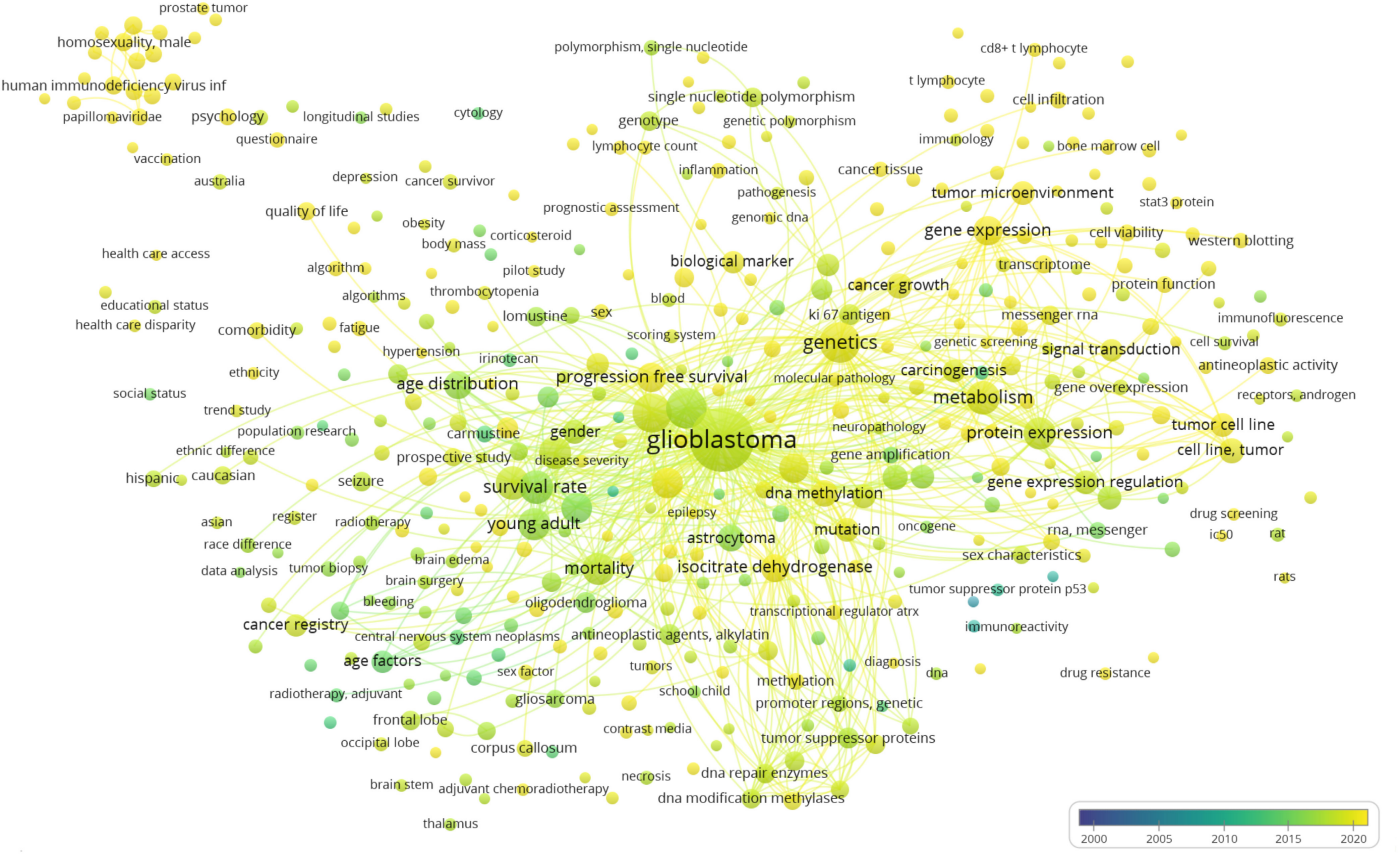
The bibliometric network map visually represents age and gender‐related studies within the glioblastoma multiforme context from 2000 to 2023. Each bubble corresponds to a specific topic or keyword, with its size indicating publication frequency and interconnections depicting relationships between topics. The color gradient, transitioning from early blue to recent yellow, signifies chronological evolution. This visualization was generated using VOS viewer (version 1.6.20, www.vosviewer.com) and utilizes scientific articles from the Scopus database.

In 2018, Minjie Tian et al. [35] utilized the Surveillance, Epidemiology, and End‐Results (SEER) database to study GBM patients who underwent surgery from 2000 to 2008. Of the 6586 identified GBM patients, 65.5% were male, which closely aligns with our findings. The study concluded that gender significantly predicts GBM risk. A 2021 study by Osawa et al. [36] investigated 137 GBM patients, with 22.6% being elderly (over 75 years old). Non‐elderly patients had a significantly longer average overall survival (15.8 months) than the elderly group (10.8 months). Similarly, non‐elderly patients had a significantly longer average progression‐free survival (9.1 months) compared to the elderly group (6.6 months). The study suggested that, for patients aged 75 and older with a Karnofsky Performance Status (KPS) below 70, considering less aggressive treatment in addition to radical resection could be a viable therapeutic option. In a 2015 analysis by Brodbelt et al. [37] involving 10 743 patients (60% males) GBM patients in England from 2007 to 2011, the average overall survival was 6.1 months, with survival rates of 28.4%, 11.5%, and 3.4% at one, two, and five years, respectively. Survival declined significantly with increasing age, from 16.2 months in the 20–44 age group to 3.2 months in those aged 70 and above. Among patients receiving maximum therapy, patients under 70 years had an average survival of 14.9 months. While maximum therapy enhanced overall survival across all age groups, individuals over 60 were less likely to receive complete combination therapy [37]. These age‐related differences align with our findings, where 35.4% of GBM patients were over 60, experiencing notably shorter mean overall survival and a lower one‐year survival rate compared to their younger counterparts. Moreover, to highlight the current trends in research related to age and gender within the context of GBM, a bibliometric analysis was performed (Figure 6).

Our study has several limitations. As a retrospective analysis, the dataset is limited to GBM patients from Guilan Province, which limits the generalizability of our findings to broader populations. Additionally, critical clinical details, such as treatment modalities and the IDH1 mutation or MGMT promoter methylation status, were unavailable. These factors are known to significantly influence survival and treatment outcomes [13]. The relatively low incidence of GBM, combined with the exclusion of a large number of patients, further impacts the generalizability of our results, and we acknowledge that these findings should be interpreted with caution. The limited patient data from each hospital also highlights the need for larger sample sizes to address potential inter‐hospital variability. Future prospective studies with more extensive datasets are necessary to better understand sex‐based differences in survival and outcomes for gliomas.

## 5 Conclusion

This multicenter study highlights the significant impact of age and gender on GBM survival outcomes. The reduced survival rates in older women suggest that hormone replacement therapies should be explored for their potential role in improving post‐menopausal outcomes. With higher survival rates in younger patients, particularly women, we recommend stratifying GBM treatment protocols by both age and gender to optimize care. Further prospective research on hormonal modulation could inform clinical guidelines, providing more personalized treatment strategies that improve survival in this challenging disease.

## Author Contributions

**Zoheir Reihanian:** conceptualization, validation, resources, project administration, writing – original draft. **Elahe Abbaspour:** writing – original draft, writing – review and editing, visualization, data curation, project administration. **Nooshin Zaresharifi:** investigation, validation, conceptualization, methodology. **Sahand Karimzadhagh:** writing – original draft, writing – review and editing, visualization. **Maral Mahmoudalinejad:** data curation, investigation, methodology, project administration. **Ainaz Sourati:** supervision, project administration, conceptualization, writing – review and editing, investigation. **Mohaya Farzin:** validation, conceptualization, methodology. **Habib EslamiKenarsari:** formal analysis, data curation, methodology, software.

## Ethics Statement

This study received approval from the Institutional Review Board at Guilan University of Medical Sciences, with the ethics approval code IR.GUMS.REC.1401.461.

## Conflicts of Interest

The authors declare no conflicts of interest.

## Data Availability

The data used and analyzed to support the findings of this study are available upon request from the editor‐in‐chief through the corresponding author.
